# Dependent-Gaussian-Process-Based Learning of Joint Torques Using Wearable Smart Shoes for Exoskeleton

**DOI:** 10.3390/s20133685

**Published:** 2020-06-30

**Authors:** Jiantao Yang, Yuehong Yin

**Affiliations:** State Key Laboratory of Mechanism System and Vibration, Institute of Robotics, Shanghai Jiao Tong University, Shanghai 200240, China; JTYang@sjtu.edu.cn

**Keywords:** dependent Gaussian process (DGP), composite kernel function, human gait, joint-torque learning

## Abstract

Estimating the joint torques of lower limbs in human gait is a highly challenging task and of great significance in developing high-level controllers for lower-limb exoskeletons. This paper presents a dependent Gaussian process (DGP)-based learning algorithm for joint-torque estimations with measurements from wearable smart shoes. The DGP was established to perform data fusion, and serves as the mathematical foundation to explore the correlations between joint kinematics and joint torques that are embedded deeply in the data. As joint kinematics are used in the training phase rather than the prediction process, the DGP model can realize accurate predictions in outdoor activities by using only the smart shoe, which is low-cost, nonintrusive for human gait, and comfortable to wearers. The design methodology of dynamic specific kernel functions is presented in accordance to prior knowledge of the measured signals. The designed composite kernel functions can be used to model multiple features at different scales, and cope with the temporal evolution of human gait. The statistical nature of the proposed DGP model and the composite kernel functions offer superior flexibility for time-varying gait-pattern learning, and enable accurate joint-torque estimations. Experiments were conducted with five subjects, whose results showed that it is possible to estimate joint torques under different trained and untrained speed levels. Comparisons were made between the proposed DGP and Gaussian process (GP) models. Obvious improvements were achieved when all DGP *r*^2^ values were higher than those of GP.

## 1. Introduction

Lower-limb exoskeletons are desired to replicate human-gait mechanics, and to be “transparent” to users [1,2]. However, no breakthrough has been made in the field of transparent human–exoskeleton interaction. In human–exoskeleton systems, a high-level controller should identify humans’ planned actions, known as motion intents, and accordingly command the robot [3]. Thus, how to determine future human motion has become a key issue. As the human gait is complex, involving activities in the nervous system, musculoskeletal dynamics, and co-operation between different joints in the lower extremity, many reported methods for human-motion-intent learning were based on pattern-recognition results for simplification [4,5,6]. Switching rules and if–then decision making were usually used for designing high-level exoskeleton controllers [7,8]. This means that they are unable to handle the evolving dynamics that is not included in preset classifications. Although various improvements on information exchange were made, there are mismatches and disparities in robots’ understanding of human-motion intents in terms of continuous joint torque, especially when a human walks at unspecified speed levels [9]. This calls for a robust model to continuously estimate joint torques for the optimization of exoskeleton assistance, which can extract the time-varying movement features and diversity of gait patterns [10,11,12].

Extensive research has been conducted on developing algorithms for estimating joint torques. Those algorithms are classified as model- or nonmodel-based strategies. The focus of model-based algorithms is to put on the estimation of joint torques by forward or inverse dynamics [13,14]. For the forward dynamics of the human neuromusculoskeletal system, most existing models are based on Hill’s work. For example, the musculoskeletal model of human lower limbs was derived for the simulation of human gait in [15]. Ao et al. proposed a surface-electromyography (sEMG)-driven Hill-type model to estimate ankle-joint torques [16]. However, as muscular contraction is a highly complex mechanochemical process including the generation of action potentials (APs), the release of myoplasmic calcium, and the final relative sliding of thin and thick filaments, the transformation from muscle activation to muscle force is not yet fully understood. Furthermore, modeling parameters may deviate from initial parameters because of fatigue during walking. In addition, the determination of muscle geometry for a living person is difficult and time-consuming. Thus, most existing modeling approaches were inaccurate and did not incorporate the time-varying capabilities of users. To address these problems, empirical models with greatly improved usability were derived by some researchers, including phenomenological [17] and semiphenomenological [18] models. Unfortunately, problems also exist with the application of empirical models, when clear biomechanical interpretations are still missing, and the model-calibration workload is heavy. Gui proposed a two-step learning strategy to estimate the active joint torques of the subject for a custom human–exoskeleton system using sEMG signals [19]. The proposed estimator could update the EMG torque model without calibrations. Experiments were only conducted for the swing phase. Therefore, it may need further investigation for the stance phase. On the other hand, the inverse-dynamics approach has been applied to estimate the resultant moment at a joint in the laboratory using an optical motion-capture system combined with force plates [20]. However, the equipment is expensive and cannot meet the requirements of daily activities. These drawbacks hinder its potential use in developing high-level controllers for walking-aid devices.

With the fast development of machine learning, there are many researchers attempting to estimate joint torques using nonmodel-based methods, including neural networks (NNs) and the Gaussian process (GP). These methods are to map the measured signals (e.g., ground reaction force, shank angular velocity, and joint angles) to the resultant moments at a joint, which can generally be formulated as a regression problem. As ankle torques are the least variable among joints of lower limbs due to the constraint by the ground, many reported algorithms are keen on the estimation of ankle-joint torques [21]. For example, an NN was adopted to estimate ankle-joint torques using a low-cost pressure insole and tendon sensor in [22]. The GP model was used to estimate ankle angles and torques at a specified walking speed using shank angular velocity and angle as the inputs [7]. This approach demonstrated superior performance and was capable of providing credibility to the established model [23]. However, estimation results on knee and hip torques remain unknown. Estimating joint torques is a highly challenging task for different levels of walking speed. A central difficulty in high-precision estimations is in determining a model that can capture dynamic information behind measured signals. As joint kinematics (e.g., angle and angular velocity) and joint torques definitely have dependencies, data fusion is recommended to explore correlations between the kinematics and torques of a joint, and to figure out time-varying movement features in the human gait. More specifically, the joint kinematics of the lower limbs are the direct description of the human gait, which can be realized by modulating the joint torques [21]. Thus, the constructed fusion model promises to offer superior performance by fusing multiple datasets. Recently, the dependent Gaussian process (DGP) was used to address the data-fusion problems [24,25]. The DGP model can be constructed by regarding the GP as filters excited with white source noise, which is a powerful mathematical tool to model various dynamic systems in terms of covariance functions [26]. It enables the exploration of deep-layer relationships between strongly coupled multisource information by considering spatial correlations with itself in each dataset and the spatial-correlation cross-datasets [27]. In addition, the statistical nature of DGP offers superior flexibility and credibility for risk-based control [23]. With these advantages, this paper presents a DGP-based data-fusion model for joint-torque estimation in the human gait. As ground reaction force (GRF) and foot motion are the direct indicators of human gait [28], measurements from wearable smart shoes were treated as inputs to the model. Joint kinematics were used for training the DGP model, and it did not need the information of joint kinematics in the prediction process. Thus, the proposed method could realize accurate predictions only by using the smart shoe, which is low-cost, nonintrusive for human gait, and comfortable to wearers. On the other hand, the design methodology of dynamic specific kernel function is presented to transfer prior knowledge of measured signals, which broadens its applications in engineering. The kernel function is the core of a GP, as it encodes prior knowledge about the dynamic system that it aims to learn [29]. Every kernel function has its suitable characteristics to model different dynamic processes [30]. The DGP with the designed composite covariance kernel could handle the evolving dynamics in human gait.

This study developed a DGP-based data-fusion model for human-lower-limb torque learning using smart shoes. The model aimed to understand the natural relationships between kinematics and torques of a joint that are dependent. The design methodology of a dynamic specific kernel function is proposed to form composite kernel functions by virtue of the sum and product constructions in which estimation bias and variances of the fused model are served as critical criteria for performance evaluation. The main contributions of this paper are as follows:

1. A soft smart shoe that is low-cost, nonintrusive for human gait, and comfortable to wearers was designed to acquire the information concerning GRF and foot motion.
2. The DGP was performed to fuse the joint kinematics and joint torques with measurements from smart shoes as the inputs. As joint kinematics are only used in the training phase, and it does not need information on joint kinematics in the prediction process, the proposed method could realize accurate estimations in outdoor activities by using only the smart shoe.
3. The designed composite covariance kernel function could achieve multiple-feature modeling at different scales, and cope with the temporal evolution of the human gait. Hence, the proposed model could extract time-varying gait patterns that were deeply embedded in the data, offering superior performance. In addition, it enabled generalized joint-torque estimations for different input types.

Experiments are also presented to demonstrate the flexibility and superior performance in learning joint torques. To the best of our knowledge, the DGP model with a composite kernel function for joint-torque estimations has not been reported before. Additionally, the proposed methods could achieve excellent performance compared with that of the GP model. 

## 2. Materials and Methods 

### 2.1. Wearable Smart Shoes

**Figure 1:**
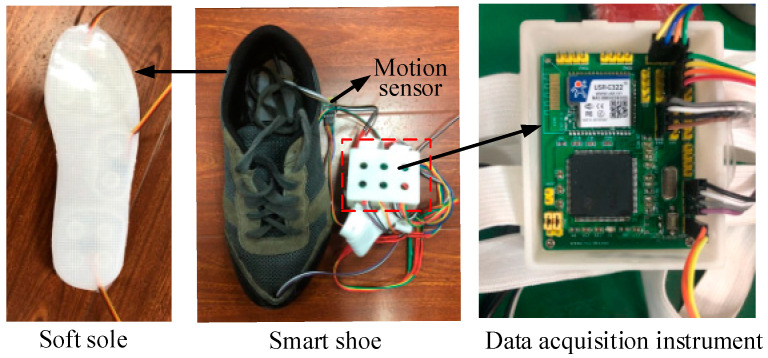
Wearable smart shoe.

In this section, we briefly introduce the smart shoe designed for exoskeletons. As shown in Figure 1, it was developed with a soft sole made of silicone rubber with Hardness Shore 35A (Ecoflex0035, SmoothOn Inc., Macungie, PA, USA), two 3D motion sensors (MPU9250, InvenSense, San Jose, CA, USA), and a self-designed data-acquisition instrument. There were three pneumatic chambers at the heel, arch, and forefoot of the sole, respectively, since the major weight is distributed on the heel and forefoot in the case of a normal gait, and pressure under the arch helps to detect the midstance phase [31,32]. A barometer (MS5637-02BA03, Measurement Specialties Inc., Fairfield, NJ, USA) was enclosed in each chamber to measure air pressure when the wearer walked on the ground. The 3D motion sensors mounted at the heel and forefoot were developed with a gyroscope, an accelerometer, and a magnetometer to provide the foot motions. A microprocessor (STM32f103, STMicroelectronics Inc., Geneva, Switzerland) and a Wi-Fi module (CC3200R1M2, Texas Instruments Inc., Dallas, TX, USA) were integrated into a printed circuit board. Air pressure (with an accuracy of ±1.5 mbar in the three pneumatic chambers, orientations (accuracy of ±0.01°) of the heel and forefoot, and angular velocities (accuracy of ±0.05°) of the heel and forefoot were acquired with a sampling rate of 100 Hz, and then transported to a personal computer by the Wi-Fi module on the data-acquisition instrument. The soft wearable shoes have numerous advantages, e.g., low cost, and being nonintrusive for human gait and comfortable to wearers.

### 2.2. DGP-Based Torque Estimation in Human Gait

Generally speaking, joint torques in the human gait are complex, involving musculoskeletal dynamics, ligament forces, and bone-on-bone forces [21]. The problem of estimating joint torques is to develop an appropriate mapping function $y_{1}=f\left(x\right)$ between input x and joint torque $y_{1}$. In the present paper, the novel smart shoes were used for measuring the system states during human walking. A set of measured data, such as $x_{l}=\left[\begin{array}{ccc} x_{1}^{} & \cdots & x_{15}^{} \end{array}\right]\in {\mathbb{R}}^{n\times 15}$, for the left shoe could be acquired, including air pressure at the heel, arch, and forefoot of the sole, three orientations, and three angular velocities of the heel along the X, Y, and Z axes, respectively; and three orientations and three angular velocities of the forefoot along the X, Y, and Z axes, respectively. Similarly, the dataset measured from the right shoe can be denoted as $x_{r}=\left[\begin{array}{ccc} x_{16}^{} & \cdots & x_{30}^{} \end{array}\right]\in {\mathbb{R}}^{n\times 15}$. Thus, the model input is $x=\left[\begin{array}{cc} x_{l} & x_{r} \end{array}\right]$, where *n* is the lag number of the inputs. DGP was performed to learn mapping function f. DGP belongs to the framework of Bayesian inference, which is fully specified by its mean and covariance function as given by Equation (1) [33]:

(1) f(x)∼GP(m(x),∑(x,x))

where m(x) is the mean function, and ∑(x,x) is the covariance function that specifies the covariance between pairs of random variables. The covariance function evaluated at pairs $x_{1}=\left[\begin{array}{ccc} x_{1}^{1} & \cdots & x_{1}^{n} \end{array}\right]$ and $x_{2}=\left[\begin{array}{ccc} x_{2}^{1} & \cdots & x_{2}^{m} \end{array}\right]$ is defined as follows:

(2) $$\Sigma \left(x_{1},x_{2}\right)={\left(\begin{array}{cccc} \varsigma \left(x_{1},x_{1}\right) & \varsigma \left(x_{1},x_{2}\right) & \cdots & \varsigma \left(x_{1},x_{m}\right)\\ \varsigma \left(x_{2},x_{1}\right) & \varsigma \left(x_{2},x_{2}\right) & \cdots & \varsigma \left(x_{2},x_{m}\right)\\ \vdots & \vdots & \cdots & \vdots \\ \varsigma \left(x_{n},x_{1}\right) & \varsigma \left(x_{n},x_{2}\right) & \cdots & \varsigma \left(x_{n},x_{m}\right) \end{array}\right)}_{n\times m}.$$

Covariance specifies which structure the learned function is likely to be, and, in turn, determines the generalization ability of the model. The squared-exponential (SE) function is the most commonly used in the field of machine learning:

(3) $$\sum \left(x_{i},x_{j}\right)=\sigma^{2}\exp\left[-\frac{1}{2}s^{T}\Lambda s\right],$$

where $s=x_{i}-x_{j}$; σ and Λ are hyperparameters defining characteristic length scales. The SE is suitable to model smooth dynamics, and can convert global correlation into local correlation.

**Figure 2:**
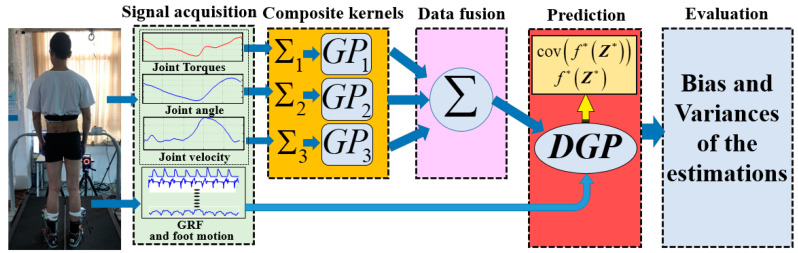
Framework of dependent Gaussian process (DGP)-based data fusion for joint-torque estimations.

Figure 2 illustrates the framework of the proposed torque-estimation procedure, including signal acquisition, the design of the composite covariance kernel functions, data fusion, prediction, and error evaluation. Information concerning GRF and foot motion is treated as the input of the model. Joint torques, angles, and angular velocity were regarded as the outputs of the model (dependent Gaussian processes are known as multioutput Gaussian processes that can be used to simultaneously handle multiple correlated outputs). The kinematics (angle and angular velocity) and torques of a joint definitely have dependencies. Hence, the fusion of multiple datasets can be formulated as a conditional estimation problem where the estimation performance of a joint torque is improved by incorporating information from the measured angle and angular velocity at the same joint. First, appropriate covariance kernel functions for the kinematics and torques of each joint should be designed in accordance to prior knowledge of the measured signals. Then, the DGP model and designed composite covariance functions are used to learn the coupling relationships of the joint kinematics and joint torques. Lastly, the estimator takes measurements from the wearable smart shoes as inputs to estimate the joint torques. Estimation bias and variances are used to qualify the reliability of the model. 

Given three measured datasets $\left(x_{1},y_{1}\right)$, $\left(x_{2},y_{2}\right)$, and $\left(x_{3},y_{3}\right)$, estimation performance is improved by learning auto-co-variance functions and cross-co-variance functions between them. This can be formulated as

(4) $$f^{*},\mathrm{cov}\left(f^{*}\right)|X^{*},x_{1},y_{1},\theta_{1},x_{2},y_{2},\theta_{2},x_{3},y_{3},\theta_{3},$$

where $x_{1}$, $x_{2}$, and $x_{3}$ are DGP inputs. In the present paper, $x_{1}$, $x_{2}$, and $x_{3}$ are the same, and all of them are equal to x; $y_{1}$,$y_{2}$, and $y_{3}$ are the joint torque, angle, and angular velocity, respectively; $\theta_{i}$ is the hyperparameter of the *i*th GP model; $X^{*}$ is an arbitrary location to be evaluated; and $f^{*}$ and $\mathrm{cov}\left(f^{*}\right)$ are the evaluated mean and covariance at $X^{*}$.

By performing DGP for joint-torque learning, a fused model can be designed by the conditional estimation of the three datasets, and it is specified in Equation (5):

(5) $$\{\begin{array}{l} f^{*}\left(X^{*}\right)=\sum \left(X^{*},X\right)\sum^{-1}\left(X,X\right)\left[y_{1},y_{2},y_{3}\right]\\ \mathrm{cov}\left(f^{*}\left(X^{*}\right)\right)=\sum \left(X^{*},X^{*}\right)-\sum \left(X^{*},X\right)\sum^{-1}\left(X,X\right)\sum \left(X,X^{*}\right) \end{array}$$

(6) $$\sum \left(X,X\right)=\left[\begin{array}{ccc} \Sigma_{11}^{Y} & \Sigma_{12}^{Y} & \Sigma_{13}^{Y}\\ \Sigma_{21}^{Y} & \Sigma_{22}^{Y} & \Sigma_{23}^{Y}\\ \Sigma_{31}^{Y} & \Sigma_{32}^{Y} & \Sigma_{33}^{Y} \end{array}\right]$$

where $\Sigma_{ii}^{Y}=\Sigma_{ii}^{U}+\sigma_{i}^{2}I$,$\Sigma_{ij}^{Y}=\Sigma_{ij}^{U}$; *i*,*j* = {1,2,3}; $\Sigma_{ii}^{Y}$ represents the auto-co-variance matrix of the *i*th dataset; $\Sigma_{ij}^{Y}$ is the cross-co-variance matrix between the *i*th and *j*th datasets, and they can be derived through the convolution integral [24]; and $\sigma_{i}^{}$ denotes the measurement noise component of the *i*th dataset. 

The covariance matrix between the evaluated point and training points is given as

(7) $$\Sigma \left(X^{*},X\right)=\left[\begin{array}{ccc} \Sigma_{i1}^{U}\left(X^{*},x_{1}\right) & \Sigma_{i2}^{U}\left(X^{*},x_{2}\right) & \Sigma_{i3}^{U}\left(X^{*},x_{3}\right) \end{array}\right],$$

where $\Sigma_{i1}^{U}$, $\Sigma_{i2}^{U}$, and $\Sigma_{i3}^{U}$ are the GPs that are evaluated given another GP; $\Sigma \left(X^{*},X^{*}\right)$ represents the covariance matrix of the location being evaluated, and it can be likewise defined.

Unlike other learning methods, estimation using DGP does not only consider spatial correlations with itself in each dataset, but also spatial correlations across datasets. The fused GP model can be constructed by regarding the GP as filters excited with white source noise [26]. The auto-co-variance and cross-co-variance functions can be computed by the convolution integral as follows:

(8) $$Y_{i}\left(s\right)=W_{i}\left(s\right)+U_{i}\left(s\right)$$

(9) $$U_{i}\left(s\right)=\int_{s}k_{i}\left(s,\tau \right)X\left(\tau \right)d\tau ,$$

where, s is the data domain; $W_{i}$ represents the stationary white noise; $k_{i}$ denotes the smooth kernel; $Y_{i}\left(s\right)$ is the estimation. Applying the convolution integral technique for SE, for example, the auto-co-variance and the cross-co-variance are specified as follows:

(10) $$\Sigma_{ii}^{U}{=K}_{f}\left(i,i\right)\pi^{\frac{d}{2}}\left|\Lambda_{i}\right|\exp\left[-\frac{1}{4}s^{T}\Lambda_{i}s\right]$$

(11) $$\Sigma_{ij}^{U}{=K}_{f}\left(i,j\right){\left(2\pi \right)}^{\frac{d}{2}}{\left|\Lambda_{i}+\Lambda_{j}\right|}^{-\frac{1}{2}}\exp\left[-\frac{1}{2}s^{T}\Lambda_{ij}s\right],$$

where $\Lambda_{ij}=\Lambda_{i}{\left(\Lambda_{i}+\Lambda_{j}\right)}^{-1}\Lambda_{j}$.

The estimation bias and variances of the fused model are served as the critical criteria to characterize the quality of the fused GP model:

(12) $$\{\begin{array}{c} \left|f^{*}-\mu \left(X^{*}\right)\right|>\mu_{0}\\ \mathrm{cov}\left(f^{*}\right)>\sigma_{0} \end{array},$$

where $\mu \left(X^{*}\right)$ is the measured value; and $\mu_{0}$ and $\sigma_{0}$ are the preset thresholds. When the deviation between estimated and expected values was greater than the preset threshold, more information was needed to achieve accurate estimation. If the estimation variance were greater than the given threshold, the estimation could be trustless. In either case, new training points should be added to provide more information. In order to meet the requirements of real-time control, the maximal length of training set *S* denoted as maxLength was preset. If *S* > maxLength, the composite covariance function should be reconstructed to reach the desired accuracy and reliability. Within this framework, hyperparameters are optimized with incoming data by using the gradient-optimization method to maximize marginal likelihood. The partial derivatives of the marginal-likelihood function with respect to the hyperparameters are acquired as follows:

(13) $$\frac{\partial \ln p\left(y|z,\theta \right)}{\partial \theta }=-1/2trace\left(\sum^{-1}\frac{\partial \sum }{\partial \theta }\right)+1/2y^{T}\sum^{-1}\frac{\partial \sum }{\partial \theta }\sum^{-1}y.$$

The basic steps of DGP for estimation are as follows (**Algorithm 1**):
**Algorithm 1** Basic steps of DGP for estimations.**Input: *Y*** = [***y***_1_
***y***_2_] (training input) ***X*** = [***x***_1_
***x***_2_] (training target)   ∑(covariance function) ***X***^✴^ (test input) **maxLength** (maximal length of training set)**Repeat:**Step 1: **If** prediction results meet critical Criteria (12).    **if** length of ***S*** ≤ **maxLength**    Added new training data ***X***_n+1_/***Y***_n+1_ to training set ***S***. Return ***S***.    **else**    Reconstruct composite covariance function until desired accuracy is reached. Return the composite covariance function.Step 2: Training hyperparameters (Equation (13)).Step 3: Update covariance matrix (Equation (6)).Step 4: Prediction with new trained hyperparameters (Equation (5)).**End**Step 5: Return $f^{*}$ (mean) and $\mathrm{cov}\left(f^{*}\right)$ (variance).

### 2.3. Design Methodology of Kernel Function

**Figure 3:**
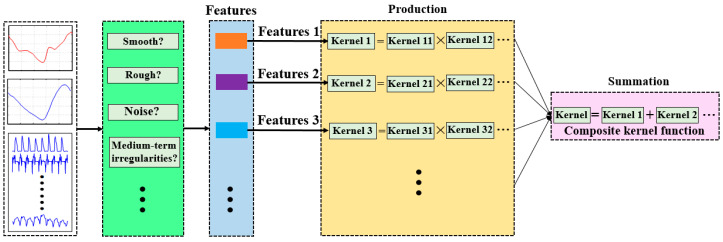
Framework of designed composite kernel function.

The kernel function is used to define the covariance. It transfers prior knowledge of the measured signals, and specifies which structure the learned function is likely to be. A befitting kernel may offer superior estimation results and determine the generalization ability of the model [34]. Commonly used base kernel functions include the squared exponential (SE), Matern class (MC), linear (LIN), white noise (WN), rational quadratic (RQ), neural network (NN), periodic (PER), and sigmoid(SIG) [29,30]. These kernel functions have different regression characteristics that can be used to model different dynamic systems. Some interpretations of properties suitable for specific system dynamics are summarized in Table 1. The human gait contains multiple features at different scales. A single covariance kernel may not be flexible enough to accurately estimate joint torques. We can combine existing base kernel functions to make a new one by virtue of the sum and product constructions. The sum of several kernels can be used to model different dynamic characteristics (periodicity, linear dynamics, nonlinear dynamics, and noise distribution), while kernels in a product way can improve the flexibility of the model [24]. The design methodology of the composite kernel function is illustrated in Figure 3. 

For instance, when we estimated the ankle-joint torque in the human gait, measurements from smart shoes were used as the inputs, and the ankle-joint torque as the output. Additionally, the ankle angle and angular velocity were incorporated to perform data fusion. First, we gathered data, and the dynamic characteristics of the signals (for example, smooth, rough, linear, nonlinear, system noise, medium-term irregularities, and periodicity) that could be inferred from the data were considered as prior knowledge. In this scenario, the leading features of the measured signals were nonlinear, containing some noise and sometimes some roughness. A summation of three different base kernels, MC, SE, and WN, was designed to model these leading features. If a certain feature was complex, only a single kernel was not enough to match the feature well. A new product kernel (e.g., MC × SE) was designed to enhance the flexibility of the DGP model. In the other scenario, for example, sEMG signals, were regarded as the inputs of the DGP model. The leading features of the sEMG signals were nonlinear random features containing some noise and roughness. A summation of NN, MC, and WN may have been the optimal choice according to the design methodology. The proposed design methodology enabled generalized joint-torque estimations for different input types.

## 3. Experiment Study 

### 3.1. Subjects

Five subjects without musculoskeletal or neurological dysfunctions gave written informed consent prior to participation in the experiments. General information about the subjects is given in Table 2.

### 3.2. Experiment Protocol

**Figure 4:**
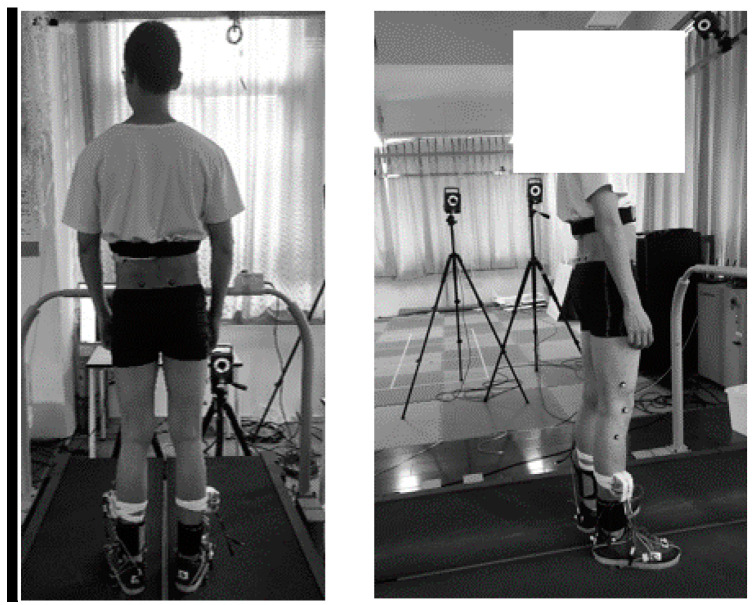
Subject and experiment setup.

As shown in Figure 4, a gait-analysis system with a treadmill and an optical motion-capture system (from Vicon Inc., Oxford, UK) were used for validating the proposed method. The treadmill had independent belts and dual force plates to measure the ground reaction force/moment for each foot. Sixteen markers (10 mm in diameter) were fixed on the subjects’ lower limbs on the following anatomical landmarks: right and left anterior superior iliac, right and left posterior superior iliac, right and left thigh, right and left knee, right and left tibia, right and left ankle, right and left heel, and right and left toe. Ten high-speed motion-capture cameras captured the markers on the lower limbs. Signals were acquired with a sampling rate of 100 Hz. After a practice phase, all subjects were required to walk on the treadmill at three walking-speed levels (0.8, 1.2, and 1.6 m/s). Two trials, denoted as Trials 1 and 2, were conducted. The subjects walked on the treadmill for 1 min and had a rest for 2 min between trails to avoid abnormal gaits due to fatigue. 

### 3.3. Data Processing

Force-plate and marker data were streamed to Nexus 2.5 software (Oxford Metrics Limited Inc. Oxford, UK), in which joint angles, angular velocities, and torques could be found. The joint torques were normalized by body weight. The dataset was divided into training and testing groups. Data of Trial 1 (relating to the three walking-speed levels mentioned above) of a specific subject were used for training the DGP model, and data from Trial 2 of that subject were used for validation. The predictor was developed using MATLAB 2015 (MathWorks, Inc., Natick, MA, USA) and run on a laptop (ThinkServer TS250 from Lenovo Ltd., Beijing, China). A combination of MC, SE, and WN was designed to model mapping function y=f(x) according to the design methodology. The MC term enabled the DGP model to regress nonlinear dynamics, whereas the additional SE allowed for the proposed method to model the local ingredient when estimations were performed near the dataset. WN was used to figure out the system noise. 

$RMS=\sqrt{\sum_{i=1}^{n}{\left(f_{i}-\mu_{i}\right)}^{2}/n}$ error was used to evaluate the estimation quality, where *n* was the total number of sample points, $f_{i}$ was the estimated value, and $\mu_{i}$ was the measured value. The root-mean-square (RMS) error contains information about the distribution of the estimated values around the expected values [8]. In addition, $r^{2}=\frac{\sum_{i=1}^{n}\left(f_{i}-\bar{\mu }\right)}{\sum_{i=1}^{n}\left(\mu_{i}-\bar{\mu }\right)}$ was computed to provide a more comprehensive understanding of the results, where $\bar{\mu }$ was the mean of measured data, $r^{2}$ values higher than 0.8 were regarded as acceptable estimations [35], and $r^{2}$ values lower than 0.6 were considered failed estimations. $r^{2}$ values and RMS errors give a comprehensive understanding of the estimation results.

## 4. Results

### 4.1. DGP Algorithm Validity

**Figure 5:**
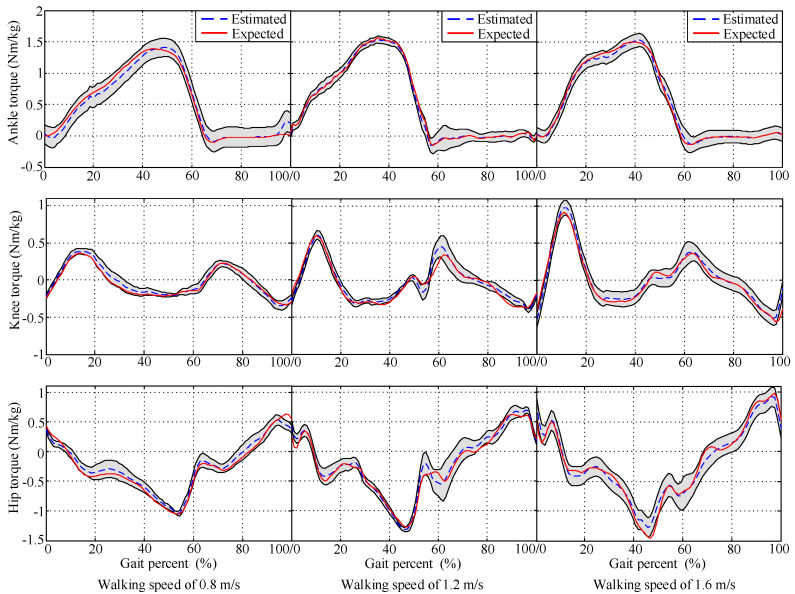
Estimation results of joint torques at walking speed of 0.8, 1.2, and 1.6 m/s, respectively; 95% confidence intervals shown in gray.

The DGP was developed in order to provide mapping from inputs to joint torques. The inputs were related to three walking-speed levels (0.8, 1.2, and 1.6 m/s). The torques were scaled to the percentage of body mass to assist in comparing different subjects. Figure 5 demonstrates a typical set of experiment results of a single step presented as percentages of the gait cycle from heel contact, where 95% confidence interval is shown in gray. With reference to Figure 5, estimation results were acceptable, with most expected values falling inside the confidence interval. In addition, torques tended to change magnitude with walking speed. As walking speed increased, the joint torques increased, since higher torques are needed at faster walking speed.

To better display the effectiveness of the proposed DGP, the *r*^2^ values for the three walking-speed levels (0.8, 1.2, and 1.6 m/s) were computed and are listed in Table 3. Only 1 out of 45 values (underlined in Table 3) was unacceptable. About 51% of the *r*^2^ values were equal to or greater than 0.90, which showed the superiority of the algorithm to some extent. In addition, the GP model was used for estimating the joint torques when the context (including kernels, datasets, and software and hardware for running the predictor) was the same as that of the DGP model during the estimation; the *r*^2^ values of GP are listed in Table 3. Only 18 values were acceptable when using GP for estimation, and 20% of the *r*^2^ values were lower than 0.6, indicating the failure of the GP model for torque learning. Comparisons were made between the proposed DGP and GP. All DGP *r*^2^ values were higher than those of GP. 

**Figure 6:**
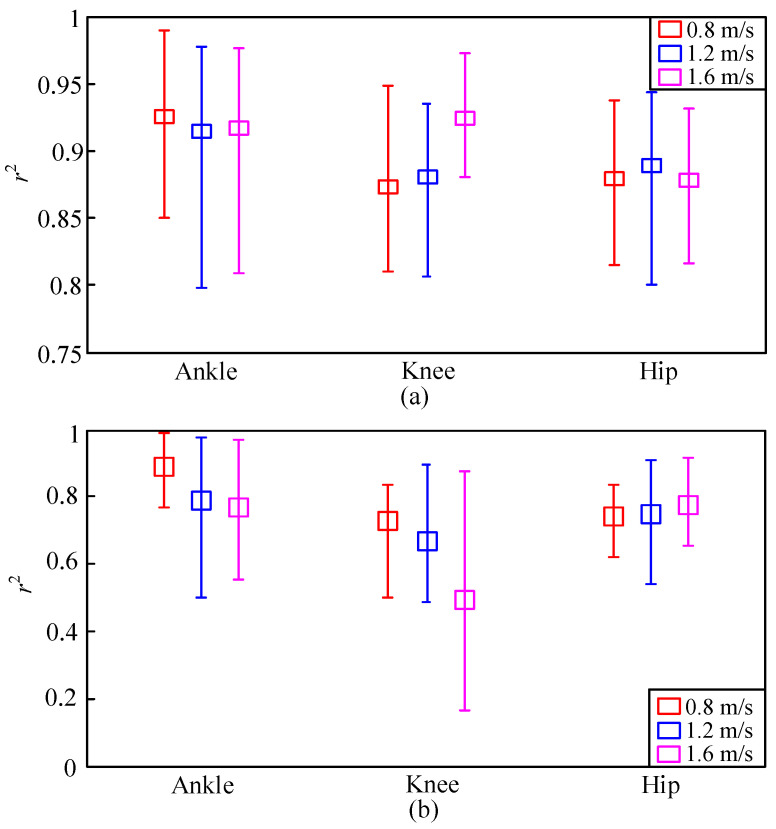
*r*^2^ values of (**a**) proposed DGP and (**b**) GP models.

The mean, maximal, and minimal *r*^2^ values using the proposed DGP model are illustrated in Figure 6a. Figure 6b shows the mean, maximal, and minimal *r*^2^ values using the GP model. The squares are the mean values of the *r*^2^ at every specific speed. The upper and lower bounds are the maximal and minimal *r*^2^ values, respectively. The mean *r*^2^ values using the proposed DGP model were all higher than 0.85 for the three walking-speed levels. The mean, maximal, and minimal values of *r*^2^ using the GP model were all lower than those of DGP model. The proposed model could, therefore, extract time-varying gait patterns and offer superior performance.

**Figure 7:**
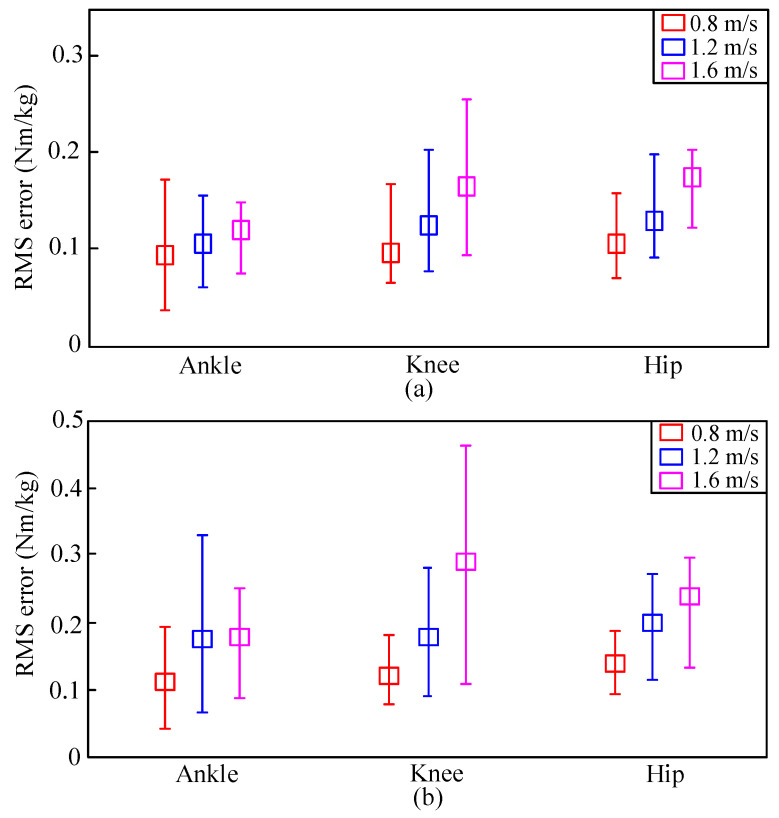
RMS errors of (**a**) proposed DGP and (**b**) GP models.

For each speed, the mean, maximal, and minimal values of RMS errors are illustrated in Figure 7. The squares show the mean of the RMS errors at every specific speed. The upper and lower bounds were the maximal and minimal RMS errors, respectively. As can be seen from Figure 7, the mean, maximal, and minimal values of DGP RMS errors were all lower than those of GP. Results indicated that the proposed DGP-based data-fusion method could understand the natural relationships from the multisource information to which it is dependent. Thus, learning unspecified human dynamics could be achieved.

### 4.2. Further Investigations

The performance of the proposed DGP model to estimate joint torques for untrained speed levels is investigated in this section. DGP was trained with input data from two of the three walking-speed levels (different combinations). Then, estimation quality was tested for another walking-speed level. For example, estimation quality was tested for a walking speed of 0.8 m/s, when the DGP was trained with input data from walking-speed levels of 1.2 and 1.6 m/s. 

**Figure 8:**
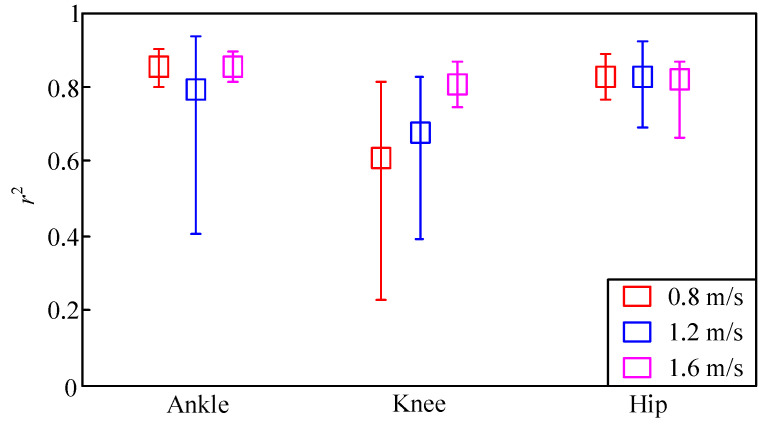
Mean, maximal, and minimal values of *r*^2^ for untrained speed levels.

**Figure 9:**
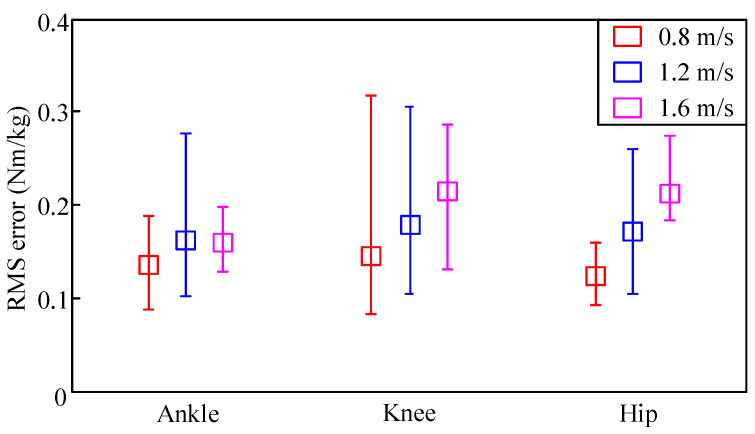
Mean, maximal, and minimal values of RMS errors for untrained speed levels.

Figure 8 demonstrates the mean, maximal, and minimal values of *r*^2^ in that case. The mean, maximal, and minimal values of RMS errors for untrained speed levels are shown in Figure 9. Almost all estimations of the GP model for untrained speed levels failed. Thus, results are not given here. As shown in Figure 8, the mean values of *r*^2^ for the three untrained walking-speed levels were all higher than 0.6, and most were higher than 0.8. The DGP model could, therefore, be used to estimate joint torques for untrained speed levels. However, the performance of the model may have declined in comparison to the trained case. This is visible for RMS errors and *r*^2^ values. By comparing Figure 6a with Figure 8, it is indicated that the mean, maximal, and minimal values of *r*^2^ for untrained speed levels were all lower than those of the trained cases. By comparing Figure 7a with Figure 9, it is indicated that the mean, maximal, and minimal RMS errors for untrained speed levels were all higher than those of the trained cases. Results were foreseeable, as gait patterns for different walking-speed levels are not necessarily the same. Meanwhile, walking-speed levels were changed with a big step size of 0.4 m/s. If we expected satisfactory results, the step size should be chosen to be smaller, e.g., 0.2 m/s. 

Although the untrained-speed result was less acceptable than in the case where there was training, the proposed model has a potential use for unspecified walking-speed levels. In the future, gait information at various walking-speed levels should be extensively incorporated in the training dataset. Then, joint torques can be estimated as well as expected for unspecified walking-speed levels.

## 5. Discussion

Human gait is definitely complex, involving the nervous and musculoskeletal systems, and fascinating co-operation between different joints and segments in the lower extremities [8]. Therefore, the human gait can be described by many different states. These different forms of information have dependencies and exist simultaneously. Taking multisource information may be the optimal choice. The GP was used to estimate the ankle torques using shank angles and angular velocities [8]. The GP model to achieve data fusion adds additional information of shank angular velocities to an existing GP model, which means that the shank angular velocities are treated as inputs to the GP model. Numerous body-attached inertial measurement units (IMUs) are required to measure shank angles and angular velocities for prediction. In this paper, the DGP-based data-fusion method was designed to explore deep-layer correlations between the kinematics (angle and angular velocity) and torques of a joint, and exploit temporal connections between measured signals. The performance of the proposed learning algorithm was slightly better than the results in [8]. Strictly speaking, comparisons between the proposed method and the algorithm reported in the recent literature are meaningless, since sensors used for signal acquisition, the datasets for training and estimating, and subject participation in the experiments were not the same. As the sEMG signal reflects human neuromuscular activities and implies muscle contraction in advance, it has been widely applied to estimate joint torques in the past few years. However, the nonstationarity and nonlinearity of physiological signals remain the main obstacles in achieving accurate joint-torque predictions. On the other hand, some researchers attempted to estimate joint torques using machine-learning methods. However, these algorithms treat the entire neuromusculoskeletal system as a black box with no biomechanical interpretations that can be formulated as a regression problem. Thus, the generalization of these models requires further investigation in the future.

One of the important merits of the DGP is that joint kinematics are used for training the DGP model, and it does not need information of joint kinematics in the prediction process. In the training phase, subjects wear the smart shoes and walk on the treadmill, which has independent belts and dual force plates to measure the ground reaction force/moment. The optical motion-capture system is used for acquire the joint kinematics. The inverse-dynamics approach is applied to obtain the resultant moment at a joint. Then, the DGP is performed to fuse the joint kinematics and joint torques with measurements from smart shoes as the inputs. It is undeniable that the training phase can be conducted in the laboratory. In the predicting phase, only measurements from smart shoes are needed. Thus, the proposed method can realize accurate estimations in outdoor activities by only using the smart shoe, which is low-cost, nonintrusive for human gait, and comfortable to wearers. 

Ankle torques are the least variable since the foot is constrained by the ground. On the other hand, knee-joint torques are the most variable of the three because the knee joint is responsible for the control of the lower extremities and torso balance [21]. In this regard, the knee torque is somewhat hard to estimate. Thus, there are many kinds of studies on estimating ankle-joint torque [7,8]. However, estimation results on the knee and hip torques remain unknown. The design methodology of a dynamic specific kernel function is proposed here. Modeling multiple features at different scales is accomplished via the design of appropriate composite covariance functions. Thus, the proposed model could extract time-varying gait patterns, and promises to offer superior performance. For the trained cases, about 98% of the total *r*^2^ values were acceptable. Unacceptable *r*^2^ values may have been the result of markers shifting during walking, which could contaminate datasets with noise. Comparisons were made between the proposed DGP and GP models. All DGP results were better than those of GP. Of GP *r*^2^ values, 20% were lower than 0.6, indicating the failure of the GP model for torque learning, while all *r*^2^ values of the proposed DGP model were higher than 0.79. Thus, correlations between joint kinematics and torques may be the key to determine the success or failure of the learning process. RMS errors contain information about the distribution of the estimations around the expected values. The distribution of RMS errors was basically irregular since the proposed DGP could figure out the deterministic structure of the system, and residual errors were random and unpredicted. 

In the case of untrained speed levels, the performance of the predictor may decline, which is foreseeable. Although estimation quality was not as good as before, the proposed DGP model could still be used to estimate joint torques for untrained speed levels with most *r*^2^ values higher than 0.8. In this study, walking-speed levels were changed with a big step size of 0.4 m/s. Thus, gait patterns for these walking-speed levels sharply differed, resulting in significant decline. In the future, the step size should be chosen to be smaller, and multisensor foot information at various walking-speed levels can be measured for training the model. Then, joint torques can be accurately estimated at unspecified speed levels for designing high-level controllers.

A balance between model precision and complexity must be reconciled, which is rarely discussed in the existing literature. The dilemma can be solved by the Bayesian information criterion [36]. In this study, prediction accuracy and computation cost were most balanced at maxLength = 500. The average time cost for estimating torques at a certain moment is about 7 ms when the predictor was developed using MATLAB 2015 and run on a laptop (ThinkServer TS250 from Lenovo Ltd., Beijing, China). Thus, it could meet the requirements of real-time control. Additionally, numerous researchers suggested various approximations to improve computation efficiency, such as the sparse-matrix method and fast matrix-vector multiplication, which are still active fields [37]. These methods can be adopted to further reduce the computation burden of the proposed DGP model. This requires more investigation in the future. Another aspect that should be considered is that any information may contain noise. In some special cases, noisy information may greatly deteriorate estimation results, although the WN kernel can remove some Gaussian white noise. Filtering is required, especially by using IMUs to measure the biomedical signals. 

## 6. Conclusions

This paper presented a DGP-based algorithm for joint torque learning in the human gait using wearable smart shoes that are low-cost, nonintrusive for human gait, and comfortable to wearers. DGP is established to perform multisource data fusion which can figure out the natural relationships among the correlated biomedical signals. The design methodology of kernel function enables multifeature modeling at different scales and can cope with temporal evolution of human gait. The experiment results showed that the proposed data-fusion model had excellent performance in the estimation of joint torques, with most expected values falling in the confidence interval; 98% of the *r*^2^ values were higher than 0.8 for the trained speed levels. Comparisons were made between the proposed DGP and GP models. Obvious improvements were achieved through the application of the proposed method over the GP model when all DGP results were better than those of GP. Although estimation quality for untrained speed levels was not as good as before, the proposed DGP model could still be used to estimate joint torques for untrained speed levels, with most *r*^2^ values higher than 0.8. In the future, multisensor foot information at various walking-speed levels could be measured for training the model. The proposed learning algorithm can be put to practical use in applications like the optimization of exoskeleton assistance, the control of active prostheses, and modulating the joint torque for humanlike robots.

## Figures and Tables

**Table 1:** Characteristics of some commonly used kernel functions. Note: SE, squared exponential; MC, Matern class; LIN, linear; WN, white noise; PER, periodic; NN, neural network; RQ, rational quadratic; SIG, sigmoid.

Kernel	Characteristics
SE	Infinitely differentiable, suitable to model smooth dynamics and kinematics.
MC	Suitable to model dynamics and kinematics with different roughness.
LIN	With linearly varying amplitude, can be used to model linear dynamics and kinematics.
WN	Gaussian white noise, can be used to model system noise.
PER	With periodic variations, suitable for periodic movements such as the standard gaits.
NN	Rapid or large variations, suitable for irregular movements with random features.
RQ	Mixture of SE with different length scales, suitable for smooth and unspecified movements.
SIG	Suitable for sudden changes, for example, sudden ground contact.

**Table 2:** Subject information used in this study (mean ± standard deviation).

Number of Subjects	Age (Years)	Height (cm)	Mass (kg)
5	26.3 ± 3.4	176.4 ± 5.3	63.3 ± 3.1

**Table 3:** *r*^2^ values relating to three walking-speed levels. GP, Gaussian process.

Subject		0.8 m/s			1.2 m/s			1.6 m/s		
		Ankle	Knee	Hip	Ankle	Knee	Hip	Ankle	Knee	Hip
A	DGP	0.8690	0.8222	0.9233	0.9491	0.8499	0.9432	0.9594	0.9731	0.8842
	GP	0.7698	0.4993	0.7427	0.9265	0.5833	0.8170	0.8565	0.3278	0.8022
B	DGP	0.9897	0.9486	0.8566	0.9773	0.9353	0.9388	0.9764	0.9574	0.9317
	GP	0.9856	0.7586	0.8256	0.9747	0.8977	0.9065	0.9675	0.8726	0.9192
C	DGP	0.9294	0.9306	0.8147	0.8845	0.9325	0.8009	0.9397	0.8810	0.8594
	GP	0.9104	0.8368	0.6702	0.5009	0.4857	0.7246	0.5536	0.3290	0.6571
D	DGP	0.9900	0.8517	0.8632	0.9619	0.8763	0.9353	0.8966	0.9062	0.8164
	GP	0.9871	0.7779	0.8384	0.8488	0.7155	0.5407	0.7123	0.1631	0.7123
E	DGP	0.8502	0.8101	0.9371	0.7983	0.8064	0.8280	0.8093	0.9024	0.8966
	GP	0.7994	0.7523	0.6183	0.6773	0.6431	0.7593	0.7411	0.7896	0.7887
